# Conservation priorities in an endangered estuarine seahorse are informed by demographic history

**DOI:** 10.1038/s41598-021-83754-4

**Published:** 2021-02-18

**Authors:** Thomas Kalama Mkare, Bettine Jansen van Vuuren, Peter R. Teske

**Affiliations:** 1 Centre for Ecological Genomics and Wildlife Conservation, Department of Zoology, University of Johannesburg, Auckland Park, Johannesburg, 2006 South Africa; 2 Centre for Aquatic Genomics, Forensics and Bioinformatics, Kenya Marine and Fisheries Research Institute, P.O. Box 81651-80100, Mombasa, Kenya

**Keywords:** Genetic markers, Ecology, Genetics

## Abstract

Historical demographic events shape genetic diversity that remains evident in the genomes of contemporary populations. In the case of species that are of conservation concern, this information helps to unravel evolutionary histories that can be critical in guiding conservation efforts. The Knysna seahorse, *Hippocampus capensis*, is the world’s most endangered seahorse species, and it presently survives in only three estuaries on the South African south coast. Factors that contributed to the species becoming endangered are unclear; additionally, the lack of information on whether the three populations should be managed separately because of potential long-term isolation hampers effective management efforts. In the present study, we reconstructed the seahorses’ demographic history using a suite of microsatellite loci. We found that the largest population (Knysna Estuary) has colonised the other estuaries relatively recently (< 450 years ago), and that its population size is comparatively large and stable. Neither of the other two populations shows signs of long-term reductions in population size. The high conservation status of the species is thus a result of its limited range rather than historical population declines. Our findings indicate that the long-term survival of *H. capensis* depends primarily on the successful management of the Knysna population, although the other estuaries may serve as reservoirs of genetic diversity.

## Introduction

Demographic events, both historical and more recent, leave long-lasting and tractable footprints in the genomes of natural populations^1^. Through reproduction, these imprints become perpetuated from ancestral to contemporary generations^2,3^. Demographic signatures can be of value in understanding the dynamics of contemporary populations^4^, and can be used to address questions concerning the causes of contemporary low diversity in species of conservation concern^5,6^. A species’ effective population size (*N*_e_) is an integral parameter in population ecology as it dictates, to a large extent, its level of genetic variation^7^. Specifically, population genetic theory predicts a positive correlation between neutral genetic diversity and effective population size, all else being equal^8,9^. Thus, when departures from this assumption are evident, a number of factors may be invoked to explain discrepancies, including an elevated evolutionary rate, selection, gene flow and changes in effective population size, including bottlenecks and founder events^7,10,11^.

Traditionally it was believed that the absence of impermeable dispersal barriers in most marine systems would automatically result in genetic homogeneity^12^. However, particularly in low-dispersal species, levels of population structure can be very high, and differing levels of neutral genetic diversity between uniquely identifiable populations are not uncommon^13,14^. In populations of endangered species, low levels of genetic diversity can have a detrimental impact on long-term species viability. In essence, genetic diversity is an indicator whether populations and/or species can adapt and persist under the influence of environmental perturbation^15^, and the maintenance of high intra-population genetic diversity is thus always beneficial^16^. Identifying potential factors that have driven the observed neutral genetic differentiation between populations is imperative and can help to guide the formulation of conservation policies.

Translocation, re-introduction or augmentation of threatened populations has been applied in a number of endangered species, with tangible success^17–19^. Despite such positive outcomes, there still exists considerable reluctance and uncertainty among conservationists towards the adoption of translocations as a conservation tool^20–23^. Indeed, when faced with severe population decline in low dispersal species, populations are often allowed to become extinct instead of facilitating the mixing of individuals from populations with unique genetic identities^24^. This reluctance stems from the fact that the mixing of populations with supposedly long histories of isolation may result in outbreeding depression and the loss of unique adaptations^22^. In addition, there is a potential risk of introducing parasites or disease-causing vectors into the recipient population^25^.

**Figure 1:**
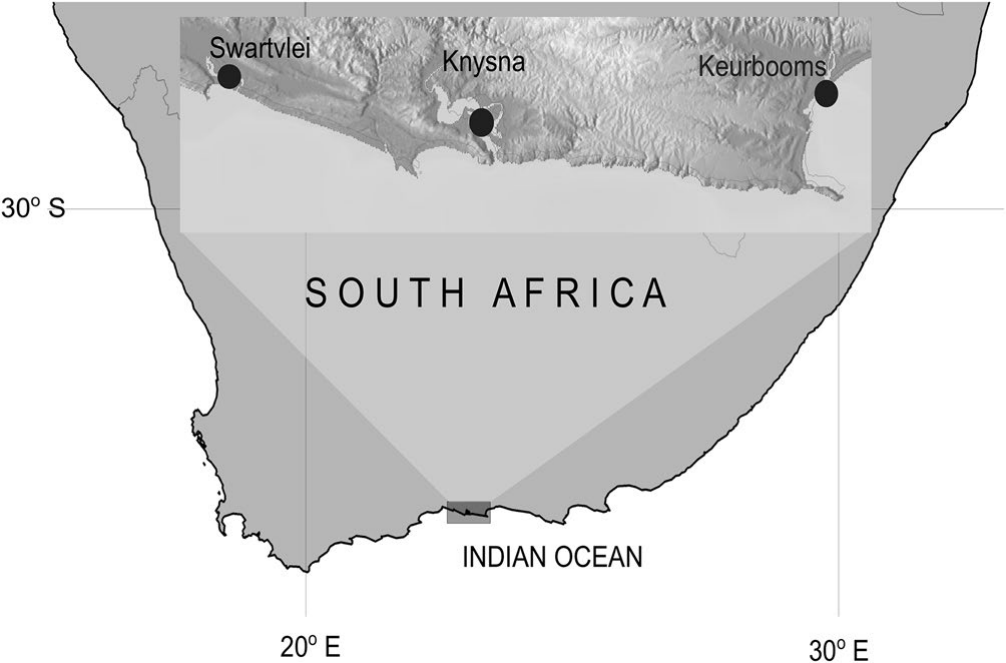
A map of the south coast of South Africa showing the three localities where *Hippocampus capensis* was sampled. The different components of the map were created separately in StepMap (www.stepmap.com) and then assembled in inkscape version 0.92 (https://inkscape.org/).

The Knysna seahorse, *Hippocampus capensis* Boulenger, 1900 is the world’s most endangered seahorse^26^. The species is endemic to South Africa and occurs in only three estuaries along the warm-temperate south coast: Keurbooms, Knysna and Swartvlei^27,28^ (Fig. 1). An analysis of mitochondrial DNA (mtDNA) sequence data conducted over a decade ago reported that each estuary harbours a genetically unique population^29^, and that the population in the Swartvlei Estuary has very low genetic diversity. These findings were confirmed in a recent study using a combination of mtDNA and a suite of hypervariable microsatellite loci^14^, which indicated that even though the Swartvlei seahorses are not inbred, levels of genetic diversity are comparable with those of captive seahorse populations^30^. However, unique genetic diversity per se is not sufficient to conclude that each population should be managed separately, as such a strategy may be to the detriment of the species. A reconstruction of the species’ demographic history, including evidence for long-term isolation of its three populations, is required to determine whether the risk of the Swartvlei population becoming extinct (to prevent outbreeding depression) is preferable to augmenting its genetic diversity.

In the present study, we used microsatellite data generated from a total of six polymorphic microsatellite loci to (a) estimate the time at which the three contemporary populations diverged from their common ancestor, (b) determine whether migration occurred subsequent to population divergence, and (c) reconstruct trends in effective population size for the three populations. The finding that the population in the Knysna Estuary recently colonised the other two estuaries provides valuable new information to help guide the process of establishing translocation programmes aimed at safeguarding the future viability of the Knysna seahorse.

## Results

### Genetic marker characteristics

The genetic characteristics of the six microsatellite loci are provided in the Supplementary Information (Table S3). Briefly, this dataset was free from laboratory artefacts such as null alleles, stuttering and large allele dropouts. Linkage disequilibrium (LD) was nonsignificant between all pairs of loci, and significant deviations from Hardy Weinberg equilibrium (HWE) were only found in the genetically impoverished Swartvlei Estuary (Table S3).

### Colonisation history of populations

**Figure 2:**
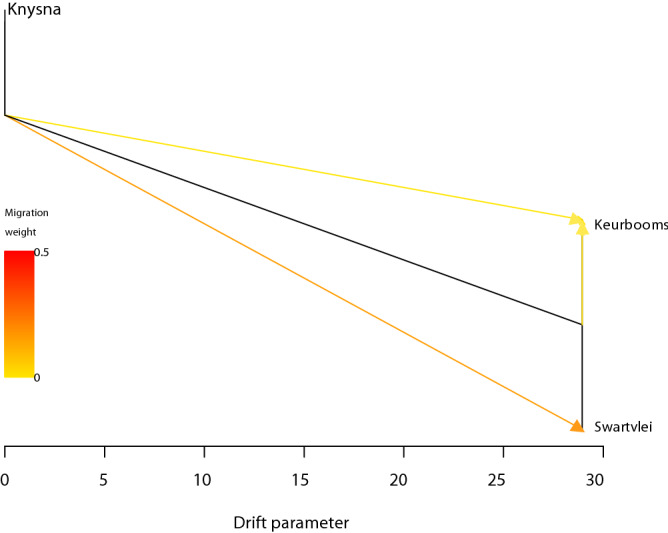
Maximum likelihood tree showing splits and gene flow between Knysna seahorse populations estimated for the microsatellite dataset using TreeMix. Arrows indicate the direction of gene flow, with migration weight indicated based on colour. The drift parameter along the x-axis gives a measure of variance in allele frequency change along each population branch.

We investigated the sequence in which the three populations were formed using the graph-based program TreeMix version 1.12^31^ (Fig. 2; see Fig. S1 for the residual fit of the maximum likelihood tree). The Knysna Estuary population is ancestral, and gave rise to the other two populations. Following initial colonization, subsequent gene flow is evident, with both the Keurbooms and Swartvlei populations receiving immigrants from the Knysna Estuary. Gene flow was higher into the Keurbooms Estuary than into the Swartvlei Estuary (Fig. 2). In addition, while the Keurbooms Estuary received migrants from the Swartvlei Estuary, there was no gene flow in the opposite direction.

### Divergence times between populations

The times at which populations diverged were estimated to test two competing hypotheses of conservation relevance: (a) genetic divergence between populations occurred during historical times, and it is possible that each population now represents a distinct evolutionary lineage; translocations should therefore be discouraged; or (b) divergence was recent, and either occurred naturally or as a result of human-mediated translocations during the recent past (decades or subsequent to European settlement); in this case, there would be no reason to discourage augmentation initiatives. The average divergence times between Knysna vs. Keurbooms, Knysna vs. Swartvlei, and Keurbooms vs. Swartvlei were estimated at 171, 174 and 83 years ago, respectively (Table 1; Fig. S2). In all cases, 95% highest posterior density (HPD) intervals ranged from a few decades ago to a few hundred years, and the hypothesis that human-mediated translocations are responsible for the existence of the two smaller populations can thus not be rejected.

**Table 1:** Population splitting times (in years) between populations of *Hippocampus capensis* estimated based on the microsatellite loci. The peak (mode) as well as the 95% highest posterior density (HPD) intervals are shown.

Parameter	Knysna vs Keurbooms	Knysna vs Swartvlei	Keurbooms vs Swartvlei
Estimate	172	174	83
HPD	37 − 448	34 − 314	11 − 342

### Effective population size

**Figure 3:**
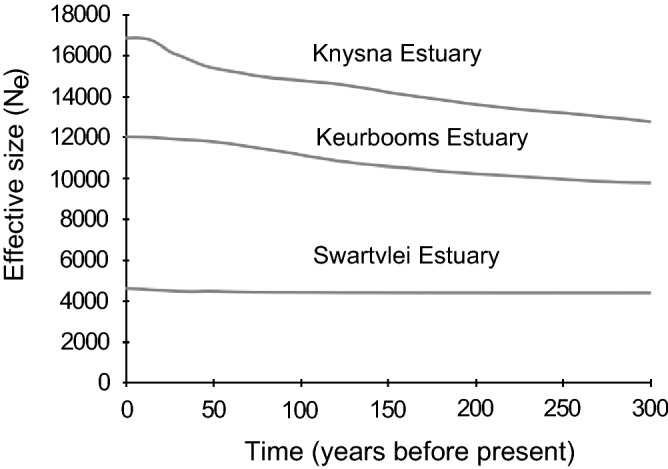
Extended Bayesian Skyline plot (EBSP) showing long-term trends in effective population size of the three populations of the Knysna seahorse.

Estimates of effective population sizes (*N*_e_) for the three populations using IMa2 indicated that the Knysna population has the largest size (*N*_e_ = 28,763), with the size of the Swartvlei population (*N*_e_ = 1780) being more than an order of magnitude smaller (Table S4, Fig. S3). The EBSPs confirmed that the Knysna population is the largest, followed by Keurbooms and lastly the Swartvlei population (Fig. 3), although the population size estimates were lower than those obtained with IMa2. The Knysna and Keurbooms populations appear to have been expanding over recent decades, whereas the size of the Swartvlei population remained stable. The contemporary estimates in *N*_e_ using the linkage disequilibrium method implemented in NeESTIMATOR corroborated the findings based on the EBSP and the IM model where the Knysna estuary had the largest *N*_e_, except that in this case, the effective population sizes in the two smaller estuaries were not different from each other (Table S5).

## Discussion

Endangered species typically have small population sizes^6^. A solid understanding of species’ demographic histories is fundamental in conservation management, particularly when species are on the brink of extirpation^6,32^. Genetic information has great utility in the conservation of endangered species^3,33,34^, as it can be applied to guide the identification of genetically impoverished populations that may require assisted recovery^35^. In many cases, the future viability of genetically impoverished populations largely depends on whether or not translocation (augmentation) aimed at increasing both population size and genetic diversity should be considered^36,37^. This is particularly true when there is little or no contemporary gene flow between geographically isolated populations.

In the present study, we reconstructed near-contemporary demographic trends to address the question whether augmentation could be considered as a practical conservation tool for the three populations of *H. capensis*. To this end, we determined the sequence of events that resulted in the formation of the populations, as well the times when they formed and how (or whether) their effective population sizes subsequently changed. Although only six microsatellite loci were incorporated in our present analyses, these were carefully selected from a larger number of published markers on the basis of being highly informative and free from laboratory artefacts. Several recent studies indicated that this number is sufficient to examine the demographic history of populations of species with limited migratory potential^36,37^.

Four findings of conservation relevance stand out. First, the Knysna population is the ancestral population of *H. capensis* and served as the source population for the other two estuaries. Secondly, these colonisation events occurred comparatively recently (between a few decades and ~ 448 years ago) and for that reason, it cannot be ruled out that they are the result of human-mediated translocations. Thirdly, there was subsequent gene flow between the three populations, in the form of either natural dispersal or the unauthorised translocation of some seahorses by humans. Lastly, the Knysna population has the largest long-term effective population size, and the Swartvlei population the smallest.

The finding that the Knysna seahorse evolved in the Knysna Estuary, most likely from a tropical marine ancestor^38^, underlines the exceptional importance of this estuary for the region’s unique biodiversity. The Knysna Estuary is the only estuarine bay on the South African south coast and has the highest biodiversity of any of South Africa’s estuaries^39,40^. In addition to housing the region’s only temperate seahorse, it is inhabited by several other rare species, including the Knysna goby, *Pandaka silvana*, which is endemic to this system^41^ and the limpet *Patella compressa*, which occurs in only one other estuarine bay^42^.

The large long-term effective population size of the Knysna population can be explained by the fact that this estuary has a large, permanently open mouth that can buffer the negative effects of freshwater floods^43^. Even though the census population sizes in the Keurbooms and Swartvlei Estuaries can at times far exceed those in the Knysna Estuary^28^, these two smaller systems occasionally experience mass mortalities^28,44^, and at times, few or no seahorses are found during diving surveys over a number of years^28^. The fact that the Knysna population does not experience such boom-and-bust cycles^28^ highlights the importance of the Knysna Estuary for the long-term survival of *H. capensis*.

We identified post-divergence gene flow between the different populations that may have been facilitated by alongshore coastal currents^45^, with the majority of migrants originating from the Knysna Estuary. However, given the young ages of the smaller populations, the possibility that these are merely the product of human-mediated rather than natural colonisation events cannot be ruled out conclusively.

## Conclusion and conservation recommendations

The question whether translocation should be considered as a conservation tool in the management plan of the endangered Knysna seahorse has remained controversial ever since the first genetic study on this species was conducted^29^. Although significant differentiation between the three systems was found using both mitochondrial and nuclear DNA markers, there was also evidence for ongoing admixture^14,29^. The finding of the present study, namely that both divergence and admixture occurred so recently that the possibility of human-mediated transport as the cause of both types of demographic events cannot be rejected, strongly argues against the idea that the different seahorse populations were isolated for prolonged periods of time and as such, must be kept isolated to prevent outbreeding depression. Our genetic data agree with anecdotal reports that unauthorised translocations have occurred repeatedly over the past decades, and possibly even earlier.

The available literature suggests that outbreeding depression can only be expected between populations with specific characteristics that include a long history of independent evolution (with evidence for morphologically distinct species or subspecies), fixed chromosomal differences, or where populations have remained in isolation for the past 500 years or more with no gene flow during this period^22^. In the present case, the different populations likely diverged no more than ~ 450 years ago, and there is clear evidence for subsequent gene flow^14^. The fact that divergence times between both population pairs are significantly different from zero indicate that despite the comparatively low number of microsatellite markers available for *H. capensis*, the present data are sufficiently informative to differentiate between divergence followed by secondary contact, as opposed to incomplete lineage sorting with alleles shared due to ancestral polymorphism^46^. Based on the present findings, we recommend the adoption of augmentation as a means of improving the population size, as well as boost genetic diversity, particularly for the population in the Swartvlei Estuary.

To this end, the Knysna population with its highest effective population size and its minimal demographic stochasticity clearly represents the most suitable donor population. Although some aspects of marina development in this estuary have had a positive impact on its seahorse population^47^, other factors typically associated with increasing human activities around estuaries, including pollution, siltation, habitat degradation, spread of aliens and diseases^48^ nonetheless constitute significant threats to the long-term survival of the Knysna population. The population size estimates of the two smaller estuaries inferred using the EBSPs are likely inflated because of post-divergence gene flow from the Knysna Estuary, since EBSP assumes no gene flow after isolation, while the IM estimates are more accurate because this method can account for post-divergence gene flow^49^. During the sampling period, seahorses were concentrated only in small areas in the two smaller estuaries, but previously published data nonetheless show that these estuaries are at least temporarily suitable to provide habitat for seahorses^27,28^. This suggests that human-mediated enhancement of their population sizes, and the repeated release of seahorses originating from the Knysna Estuary, should be considered as a management tool to preserve as much genetic diversity as possible in the event that the Knysna population experiences a significant and prolonged decline. Despite lack of evidence for boom-and-bust cycles in the Knysna Estuary, careful monitoring of the population size is required to ensure that translocations take place during periods when this population has a particularly large size. In addition to translocations, conservation efforts in the estuaries should also consider protecting the available suitable habitats of the seahorse to improve the species’ chances of survival. Overall, the ecological health of the Knysna Estuary is deteriorating^50^ and the smaller estuaries experience mass mortalities, suggesting that translocations will be most successful when environmental conditions in all three estuaries are ideal. Without a secure habitat, translocation will not benefit the species in the long term.

## Methods

### Data acquisition

We retrieved and reanalysed recently generated microsatellite data of 91 individuals (Keurbooms: n = 25; Knysna: n = 41 and Swartvlei: n = 25)^14^. The microsatellite data used was for a total of six variable loci which were selected from a total of 15 which were originally amplified (Table S2). The PCR amplification was done through a post-PCR multiplexing procedure where each locus is PCR amplified in isolation, but later the PCR products from different loci are mixed prior to genotyping. While each of the 15 loci was able to amplify, nine of these loci were associated with problems, including presence of multiple peaks, which is a signal for stuttering (i.e. *Hca*µ39, *Hhip*5 and *Hhip*6), poor amplifications where only a limited number of samples of the species were able to amplify (*Hgut*4) or the second allele being inadequately amplified, a signal for presence of large allele dropout (*Hhip*2), and some loci being invariable where one or two alleles were present (*Hca*µ25, *Hhip*9, *Hhip*10, *Han03*)^14^. The remaining six loci were found to be free from the above problems and were thus retained for subsequent analyses (Table S2). The genotyped samples from the three estuaries (Fig. 1; Table S1) were obtained during the austral summer of 2014, and were collected with permission from CapeNature (Keurbooms Estuary, permit no. CN44-59–7694) and South African National Parks (SANParks) (Knysna and Swartvlei estuaries). Additionally, animal handling procedures were approved by the Ethics Committee of the University of Johannesburg, South Africa (reference no. 2014–05-03/Teske). All animal handling procedures were performed in accordance with the relevant guidelines and regulations.

### Ethics statement

Samples were collected with permission from the CapeNature (Keurbooms Estuary) and the South African National Parks (SANParks) (Knysna and Swartvlei estuaries). Animal handling procedures were approved by the Ethics Committee of the University of Johannesburg, South Africa.

## Data analyses

### Colonisation history of populations

We investigated the sequence in which the three populations were formed (colonisation history) by using the graph-based program TreeMix version 1.12^31^. This programme was also used to investigate whether migrations between populations occurred post-divergence. The analysis was performed firstly by trying to identify the maximum possible number of migration events (m) between populations, starting with m = 1 to a maximum of m = 6 (for three populations, each donating or receiving migrants). The final analysis was then performed with the maximum migration events available in the dataset specified at m = 3, with default settings used for all other parameters.

### Splitting times between populations

The times at which populations diverged from each other were estimated using the coalescent based, Markov Chain Monte Carlo (MCMC) program IMa2^49^. IMa2 implements the isolation with migration (IM) model^51^, and estimates populations splitting times while taking into account changes in effective population size and post-divergence gene flow. The IMa2 analyses were performed on population pairs (i.e. Knysna vs. Keurbooms, Knysna vs. Swartvlei, and Keurbooms vs. Swartvlei) to obtain a posterior estimate of the splitting time (*t*_0_) since the two populations split from their common ancestor, including the ancestral and contemporary estimates of effective population sizes (*N*_e_). The pairwise approach is recommended when limited data are available^49^. The mutational splitting time *t* is given as *t* = *T*/u, where *T* is the time since common ancestry in generations and u is the geometric mean of the loci’s mutation rates per year. Knysna seahorses have a generation time of approximately one year^52^. Prior parameters for the population size, spitting times and migrations and two heating parameters (*h*a = 0.99 and *h*b = 0.7) were identified after a series of test runs. A burnin period of 2 × 10^6^ generations with a total of 150 heated chains (hn) and a geometric heating scheme (hfg) were specified. A teleost-specific mutation rate of 5.56 × 10^–4^ locus^-1^ year^-1^ was used, with a 95% confidence range of 1.52 × 10^–4^ to 1.63 × 10^–3^ ^53^. The analysis was repeated three times, with each run using a different starting seed. Convergence of the analyses was confirmed by an effective sample size (ESS) greater than 200.

### Effective population size

We estimated both point estimates and long-term trends in *N*_e_ for each of the three populations. Point estimates were generated using both the coalescent method implemented in the IMa2 programme (with programme settings as described above) and the linkage disequilibrium approach^54^ implemented in NeESTIMATOR version 2^55^. To run NeESTIMATOR, we selected a monogamic mating system with a critical value of 0.02 to exclude rare alleles. The long-term trends in *N*_e_ for each of the three populations were assessed by reconstructing Extended Bayesian Skyline Plots (EBSP)^56^, using BEAST version 1.7.5^57^. GenAlEx version 6.5^58^ and PGDSpider version 2.0.7.2^59^ were used for infile preparation. For each population, we linked the substitution models, but unlinked the clock models. Under the substitution sites model, we used equal rates, linear mutation bias and the two-phase* model options. Under the strict clock model, an average mutation rate with 95% confidence intervals was used^54^. The analyses were run by specifying a chain length of 1.8 × 10^8^, with parameters logged every 2 × 10^3^ iterations. The log files were analysed in Tracer v1.6^60^ to ensure that the program was run for sufficiently long for the Markov chains to reach convergence.

## Supplementary Information

Supplementary Information.
