# Supplementary material for: Conservation priorities in an endangered estuarine seahorse are informed by demographic history
Source: Sci Rep. 2021 Feb 18;11:4205. doi: 10.1038/s41598-021-83754-4 (PMC7892823; doi:10.1038/s41598-021-83754-4)
Supplement: Supplementary file 1 — Supplementary Information. [file 41598_2021_83754_MOESM1_ESM.pdf]

SUPPORTING INFORMATION

Conservation priorities in an endangered estuarine seahorse are informed by demographic history

Thomas Kalama Mkare[1,2]*, Bettine Jansen van Vuuren[1], Peter R. Teske[1]

[1]Center for Ecological Genomics and Wildlife Conservation, Department of Zoology, University of Johannesburg, Auckland Park 2006, Johannesburg, South Africa

[2]Centre for Aquatic Genomics, Forensics and Bioinformatics, Kenya Marine and Fisheries Research Institute, P.O. Box 81651-80100, Mombasa, Kenya

Table S1 Description of sampling locations and sample sizes obtained from each locality

Locality	Latitude	Longitude	*n*
Keurbooms	34° 2'12.11"S	23°23'9.10"E	31
Knysna	34°4'49.61"S	23°3'38.49"E	43
Swartvlei	34°0'47.42"S	22°45'59.95"E	25
Total	—	—	99

Table S2 Description of the 15 microsatellite loci that were PCR amplified and genotyped in the present study. The number of alleles, including observed and expected heterozygosity are those of reported by the original authors.

Locus name	Accession number	Repeat motif	No. alleles	H_{O}	H_{E}
*Hca*µ8[1]	DQ889235	$(CA)_{18}$	15	0.76	0.88
*Hca*µ10[1]	DQ889236	$(TTTC)_{6}$CTTC $(TTTC)_{12}(TTCC)_{2}$	15	0.68	0.89
*Hca*µ25[1]	DQ889241	$(TG)_{14}$	5	0.52	0.53
*Hca*µ39[1]	DQ889249	(GT)10	3	0.32	0.39
*Hhip*1[3]	–	(ATCT)42	26	1.00	0.94
*Hhip*2[3]	–	(ATCT)19	22	0.92	0.93
Hhip3[3]	–	(TCTA)22TCCA(TCTA)9	18	0.96	0.92
*Hhip*4[3]	–	(AGAT)30	17	0.92	0.91
*Hhip*5[3]	–	(CT)10CC(CT)31	19	0.88	0.92
*Hhip*6[3]	–	(TC)27	17	0.83	0.92
*Hhip*7[3]	–	(TAGA)42	30	0.96	0.95
*Hhip*9[3]	–	(GA)20TACAG(TAGC)4(TAGA)22	22	0.90	0.94
*Hhip*10[3]	–	(TC)16GTCACTCTC(TCTA)28	22	0.96	0.93
*Hgut*4[3]	–	(GATA)25	16	0.92	0.89
*Han*03[2]	–	(GT)43	22	0.949	0.952

Presence of genotyping errors such as null alleles, stuttering and large allele dropouts, were investigated using MICROCHECKER version 2.2.3[4]. Deviations from Hardy Weinberg equilibrium (HWE) and Linkage disequilibrium (LD) were investigated using GENEPOP version 44.2 on the Web[5,6]. HWE was investigated both at locus and locality level by specifying 10^4 dememorizations, 10^3 batches and 10^4 iterations per batch. It is widely accepted that HWE deviations due to homozygote excess can originate from other sources other than selection alone, such as genotyping errors and inbreeding. Presence of inbreeding in the dataset was investigated by estimating the inbreeding coefficient F_{IS} using GENETIX version 4.05[7]. The usefulness of the GENETIX program is that it provides 95% confidence intervals (CIs). When there is a zero between the CI, it means there is no evidence for inbreeding or outbreeding even when F_{IS} itself is positive or negative. The unbiased expected (H_E) and observed heterozygosity (H_O)[8] were calculated using MICROSATELLITE TOOLKIT version 3.1[9]. Allelic richness (i.e., the number of alleles per population) were calculated using the rarefaction method[10] implemented in HP-RARE version 1.1[11].

Table S3 Description of the six microsatellite loci analysed in the present study, including number of alleles (N_A), allelic richness (A_R), observed (H_O) and expected heterozygosity (H_E), and inbreeding coefficient (F_{IS}). These indices were generated for the six loci using standard procedures[12]

Locus	Index /statistic	Keurbooms (n= 25)	Knysna (n= 41)	Swartvlei (n= 25)
*Hhip*1	$\boldsymbol{N_A}$	5.00	7.00	4.00
	A_R	4.83	4.97	4.00
	H_O	0.61	0.57	0.11
	H_E	0.68	0.53	0.20
	F_{IS} (95% CI)	0.11 (-0.21- 0.38)	-0.08 (-0.34 - 0.16)	**0.48** (-0.08 - 1.00)
*Hhip*3	$\boldsymbol{N_A}$	15.00	22.00	9.00
	A_R	13.62	16.42	8.62
	H_O	0.88	0.94	0.78
	H_E	0.88	0.93	0.82
	F_{IS} (95%)	0.0103 (-0.17 - 0.17)	-0.0206 (-0.10 - 0.04)	0.05 (-0.16 - 0.23)
*Hhip*4	$\boldsymbol{N_A}$	11.00	15.00	9.00
	A_R	10.44	11.23	8.75
	H_O	0.95	0.84	0.55
	H_E	0.83	0.80	0.54
	F_{IS} (95%)	-0.15 (-0.29 to -0.05)	-0.05 (-0.19 to - 0.08)	-0.02 (-0.24 to - 0.16)
*Hhip*7	$\boldsymbol{N_A}$	5.00	3.00	5.00

	A_R	4.48	2.73	4.23
	H_O	0.39	0.45	0.16
	H_E	0.53	0.46	0.19
	F_{IS} (95%)	0.27(-0.12 - 0.60)	0.02 (-0.26 - 0.28)	0.17 (-0.08- 0.47)
*Hcaμ*10	N_A	12.00	14.00	9.00
	A_R	11.56	11.87	8.73
	H_O	0.87	0.94	0.71
	H_E	0.90	0.88	0.82
	F_{IS} (95%)	0.04 (-0.13 - 0.17)	-0.07 (-0.17 - 0.01)	**0.14** (-0.10 - 0.35)
*Hcaμ*8	N_A	13.00	17.00	8.00
	A_R	12.32	12.22	7.74
	H_O	0.83	0.72	0.84
	H_E	0.85	0.88	0.84
	F_{IS} (95%)	0.03 (-0.15 - 0.18)	**0.18** (0.02 - 0.32)	0.00 (-0.18 - 0.17)
Combined loci	N_A	10.17	13.00	7.33
	A_R	9.54	9.99	7.01
	H_O	0.75 ± 0.04	0.74 ± 0.03	0.52 ± 0.04
	H_E	0.78 ± 0.06	0.75 ± 0.08	0.57 ± 0.13
	F_{IS} (95%)	0.03 (-0.08 - 0.10)	0.00 (-0.08 - 0.05)	**0.08** (-0.04 - 0.16)

Loci and/or populations that deviated from HWE are indicated by bold letters at the F_{IS} coefficients.

Table S4 Contemporary effective population size of the three populations estimated under the isolation with migration (IM) model.

	Knysna		Keurbooms		Swartvlei	
	Θ	N_e	Θ	N_e	Θ	N_e
Estimate	3.998	28763	1.006	7237	0.248	1780
HPD95Lo	1.126	8101	0.254	1827	0.086	615
HPD95Hi	3.998	28763	3.142	22604	1.028	7395

Table S5 Contemporary effective population size of the three populations estimated using NeESTIMATOR.

	Knysna		Keurbooms		Swartvlei	
	Parametric	Jackknife	Parametric	Jackknife	Parametric	Jackknife
Estimate	7825	7825	40	40	39	39
95% CI Low	289	142	28	22	24	13
95% CI High	Infinite	Infinite	64	106	81	Infinite

Supplementary Figure

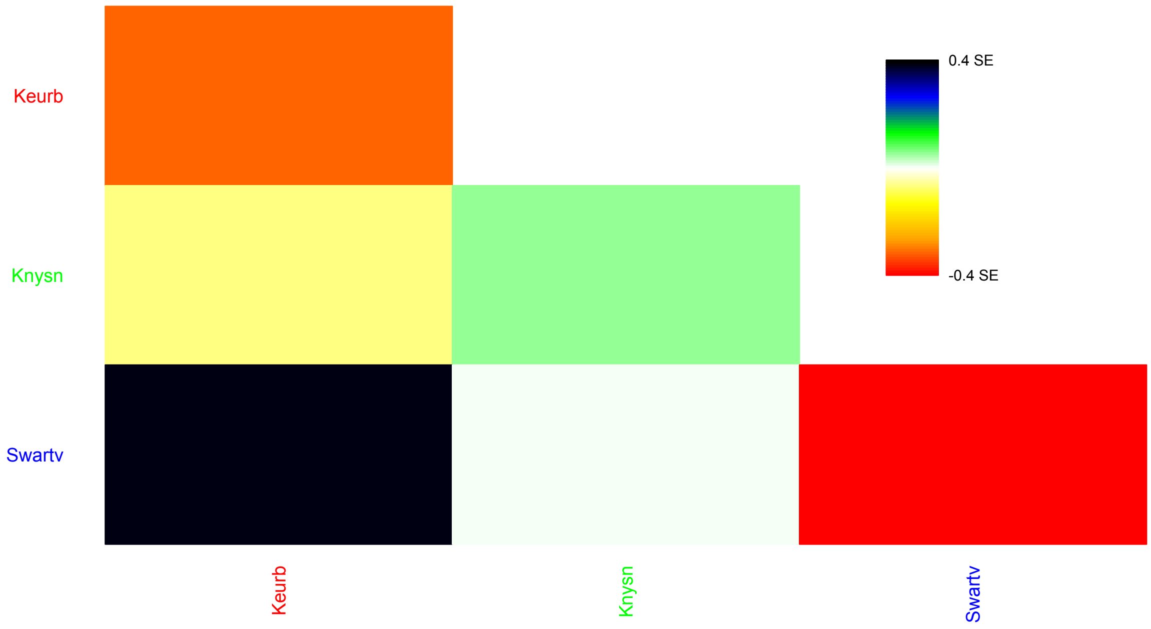

Fig. S1 TreeMix analysis showing maximum likelihood residuals showing the fitting of the model to the data.

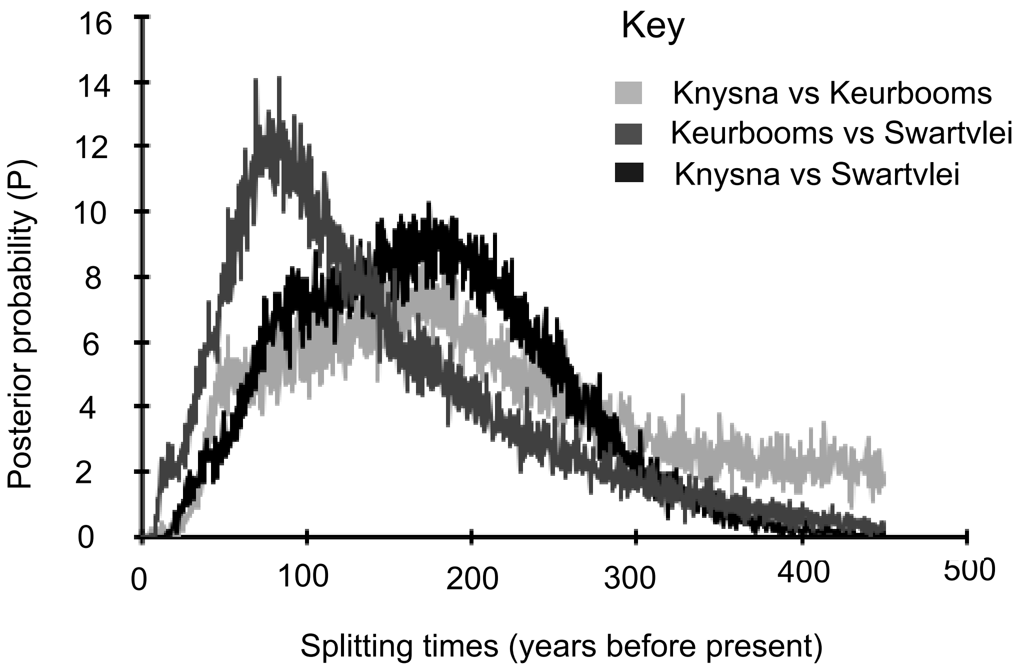

Fig. S2 Posterior probability density curves generated using the isolation with migration (IM) model showing splitting times between population pairs. The Knysna vs. Keurbooms curve (light grey) did not return to zero, so it is not possible in this case to determine the upper highest posterior density (HPD) value.

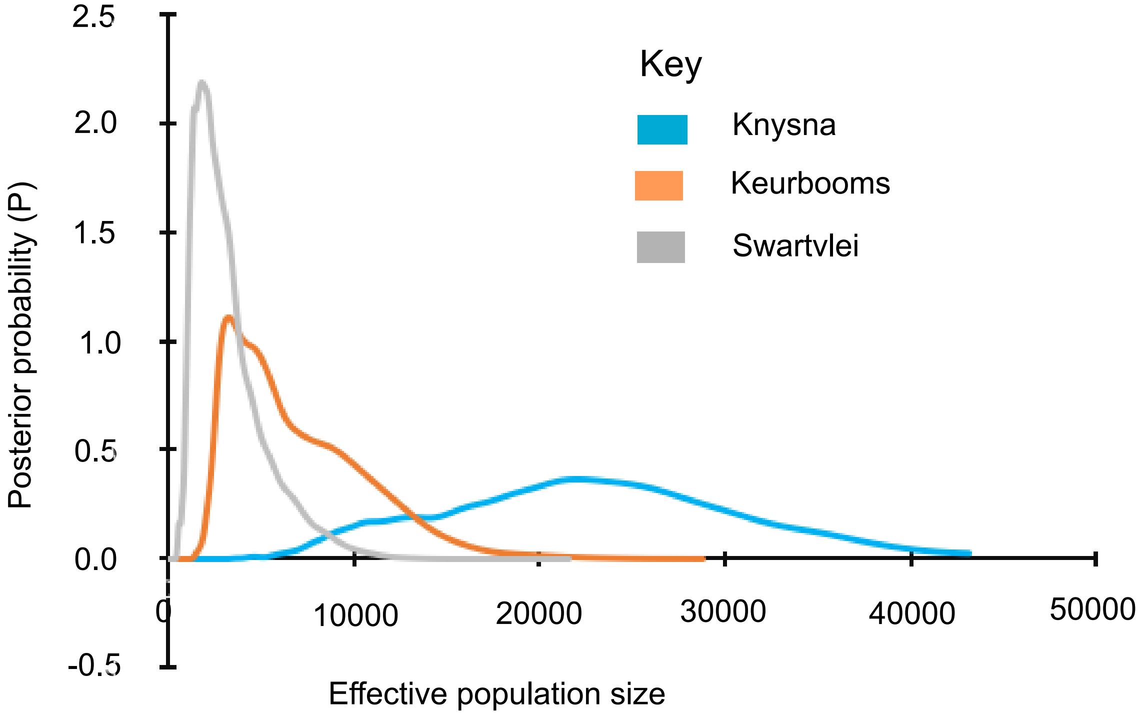

Fig. S3 Contemporary effective size estimated for each of the three populations using the isolation with migration (IM) model.

References

1. Galbusera, P.H.A., Gillemot, S., Jour, P., Teske, P.R., Hellemans, B. & Volckaert, F.A.M.J. Isolation of microsatellite markers for the endangered Knysna seahorse *Hippocampus capensis* and their use in the detection of a genetic bottleneck. Mol Ecol Notes 7, 638–640 (2007).
2. Jones, A.G., Kvarnemo, C., Moore, G.I., Simmons, L.W. & Avise, J.C. Microsatellite evidence for monogamy and sex-biased recombination in the Western Australian seahorse *Hippocampus angustus*. Mol Ecol 7, 1497–1505 (1998).
3. van de Vliet, M.S., Diekmann, O.E. & Serra˜o, E.T.A. Highly polymorphic microsatellite markers for the short-snouted seahorse (*Hippocampus hippocampus*), including markers from a closely related species the long-snouted seahorse (*Hippocampus guttulatus*). Conserv Genet Resour 1, 93–96 (2009).
4. van Oosterhout, C., Hutchinson, W.F., Wills, D.P.M. & Shipley, P. MICROCHECKER: software for identifying and correcting genotyping errors in microsatellite data. Mol Ecol Notes 4, 535–538 (2004).
5. Raymond, M. & Rousset, F. genepop, version 1.2: population genetics software for exact tests and ecumenicism. J Hered 86, 248–249 (1995).
6. Rousset, F. Genepop'007: a complete reimplementation of the Genepop software for Windows and Linux. Mol Ecol Resour 8, 103–106 (2008).
7. Belkhir, K., Borsa, P., Chikhi, L., Raufaste, N. & Bonhomme, F. Genetix 4. 02, logiciel sous Windows^{TM} pour lagntique des populations. Laboratoire génome, populations, interactions: CNRS UMR 5000, Université de Montpellier II, Montpellier, France (2001).

8. Nei, M. Molecular Evolutionary Genetics, Columbia University Press, New York, NY, USA (1987).
9. Park, S.D.E. The Excel Microsatellite Toolkit (v3.1). Animal Genomics Laboratory, UCD, Ireland (2001).
10. Kalinowski, S.T. Counting alleles with rarefaction: Private alleles and hierarchical sampling designs. Conserv Genet 5, 539–543 (2004).
11. Kalinowski, S.T HP-RARE 1.0: a computer program for performing rarefaction on measures of allelic richness. Mol Ecol Notes 5, 187–189 (2005).
12. Mkare, T.K., van Vuuren, B.J. & Teske, P.R. Conservation implications of significant population differentiation in an endangered estuarine seahorse. Biodivers. Conserv. 26, 1275–1293 (2017).
